# Evaluation of the splenic Doppler resistive index as a hemodynamic variable and its association with mortality in sepsis: A prospective cohort study

**DOI:** 10.1097/MD.0000000000047433

**Published:** 2026-01-30

**Authors:** Tatiana Buitrago-González, Claudia Poveda-Henao, Gina Montaño-Padilla, Jenny Garzón-Ruiz, Diego Jaramillo-Hernández, Henry Robayo-Amortegui

**Affiliations:** a Critical Care Resident, Fundación Clínica Shaio, Bogotá D.C., Colombia; b Department of Critical Care Medicine, Extracorporeal Life Support Unit, Fundación Clínica Shaio, Bogotá D.C., Colombia; c School of Medicine, Universidad de La Sabana, Chía, Colombia; d Clinical Research Center, Fundación Clínica Shaio, Bogotá D.C., Colombia; e Department of Radiology, Fundación Clínica Shaio, Bogotá D.C., Colombia; f Facultad de Medicina, Doctorado en Ciencias Clínicas, Universidad de La Sabana, Chía, Cundinamarca, Colombia.

**Keywords:** critical care, Doppler ultrasound, hypoperfusion, intensive care unit, mortality, sepsis, shock, splenic Doppler resistive index (SDRI)

## Abstract

This study aims to estimate the association between the splenic Doppler resistive index (SDRI) and in-hospital mortality in patients with sepsis. A prospective analytic cohort study was conducted on 109 consecutive adult patients with sepsis or septic shock, admitted to the emergency resuscitation room of a 4th-level referral center in Bogotá, Colombia, between November 2023 and October 2024, until the required statistical sample size was reached. A total of 109 consecutive adult patients (aged ≥18 years) with a diagnosis of sepsis or septic shock, based on the Sepsis-3 definition, were admitted to the emergency resuscitation room. Exclusion criteria included pregnancy, breastfeeding, inability to perform an apnea maneuver for Doppler measurement, severe hyperactive delirium, abdominal conditions limiting ultrasound access, severe peripheral arterial disease, disorders affecting hepatosplanchnic circulation, and permanent cardiac arrhythmias. The SDRI was measured within the 1st 6 hours after admission to the emergency resuscitation unit. The principal investigator received standardized training from the affiliated radiologist. Additional clinical variables were simultaneously recorded using a standardized data collection tool, including demographic characteristics, vital signs, site of infection, laboratory tests, blood culture, capillary refill time, Glasgow Coma Scale, and mottling score. In sepsis, SDRI of >0.7 showed lower survival from day 0 to day 38 of hospitalization. Thus, an association between SDRI of >0.7 and in-hospital mortality is suggested. Further studies are required to confirm these findings.

## 1. Introduction

Sepsis is the physiological, pathological, and biochemical dysregulation of the normal infection response process, leading to life-threatening organ dysfunction.^[1]^ Septic shock is characterized by cellular, metabolic, and circulatory abnormalities that require vasopressors to maintain a mean arterial pressure (MAP) above 65 mm Hg. It is characterized by the presence of hyperlactatemia (>2 mmol/L) and the absence of hypovolemia.^[1]^ The Sepsis-Related Organ Failure Assessment (SOFA) score is a validated tool used to quantify the extent of organ dysfunction in patients with sepsis and to predict clinical outcomes. An increase of ≥2 points in the SOFA score is a key criterion in the Sepsis-3 definition for diagnosing sepsis.^[2]^ Annually, 48.9 million cases of sepsis and 11 million associated deaths are reported.^[3]^ Furthermore, 85% of fatal cases occur in low- and middle-income countries.^[3]^ Multiorgan failure remains the primary cause of death in these patients^[2]^; therefore, early diagnosis is crucial for improving clinical outcomes.

Despite the emphasis on early diagnosis to improve clinical outcomes in sepsis, traditional perfusion markers present significant limitations.^[4]^ The use of macrodynamic variables often leads to late detection of hypoperfusion. These variables include MAP, which is also a resuscitation target in sepsis.^[1]^ Cutaneous variables (capillary filling and mottling score) have several limitations, such as subjectivity (they depend on the observer), difficulty in assessment in dark skin, and the need for standardized protocols for their measurement.^[5]^ Additionally, they vary according to sex, age, temperature, and environmental and skin light.^[5]^ However, limitations associated with microdynamic variables are mostly associated with the difficulty in generalizing the functionality of each microcirculatory bed, as each one has a different physiology. One of the most commonly used variables is lactate, whose values change in renal and hepatic dysfunction and/or the use of medications^[6,7]^ (Table S2, Supplemental Digital Content, https://links.lww.com/MD/R292).

Given these limitations, alternative approaches such as point-of-care ultrasound have gained relevance as noninvasive, accessible, and early diagnostic tools for assessing visceral perfusion, particularly in splanchnic organs like the kidneys, liver, and spleen.^[8–10]^ These territories often exhibit perfusion impairment before the onset of macrohemodynamic signs.^[9,10]^ Therefore, the splenic Doppler resistive index (SDRI) and renal Doppler resistive index have been proposed as potential markers of early organ dysfunction and hypoperfusion in shock states.^[9–11]^ The SDRI enables the assessment of distal vascular resistance by measuring red blood cell flow, derived from time-velocity Doppler spectra.^[12]^ It represents a viable tool for the early detection of hypoperfusion in sepsis, supported by the biological plausibility that the splanchnic circulation serves as a physiological reservoir in states of circulatory compromise.^[5,6,13–16]^ The prioritization of blood flow to vital organs results in an increase in flow resistance, directly affecting SDRI values.^[13]^ Thus, elevated SDRI values may serve as an early marker of hypoperfusion.

The main objective of this study was to estimate the association between SDRI and in-hospital mortality in patients with sepsis.

## 2. Methods

This study prospectively included 109 consecutive adult patients admitted to the emergency resuscitation room of a 4th-level referral center in Bogotá, Colombia, between November 2023 and October 2024. Patients were diagnosed with sepsis, defined as an increase of at least 2 points in the SOFA score and suspected infection or septic shock, defined as hypotension and hyperlactatemia >2 mmol/L despite volumetric resuscitation requiring vasopressor administration to maintain MAP of ≥65 mm Hg, in accordance with the Sepsis-3 definition.^[2]^

Patients aged ≥18 years with a diagnosis of sepsis or septic shock admitted to the emergency unit for resuscitation were included (according to the Third International Consensus Definitions for Sepsis and Septic Shock) in this study. Exclusion criteria were as follows: pregnancy; breastfeeding; inability to perform apnea for 3 to 5 seconds for ultrasound measurement; severe hyperactive delirium; morbid obesity and a body mass index of >40; severe abdominal distension or high intra-abdominal pressure upon admission to resuscitation; and history of severe peripheral arterial disease, splenectomy, cirrhosis, permanent cardiac arrhythmias, tension ascites, and severe abdominal wall edema.

In bias control, Cox regression was performed, and the exclusion criteria that limit Doppler measurement, were emphasized.

The sample size was calculated using the formula:


$$\begin{array}{l} n=\frac{Z^{2}\times P\times \left(1-P\right)}{d^{2}}=66 \end{array}$$

where the sepsis mortality prevalence *P* was 30%. The study protocol, ultrasound-based monitoring, including Doppler assessment, was an essential part of the routine diagnostic evaluation and standard management of critically ill patients upon admission. Given these considerations, informed consent was not requested. This approach was approved jointly by the Fundación Clínica Shaio Ethics Committee; only verbal assent from the patients was considered. Additionally, all patients admitted to Fundación Clínica Shaio grant their general consent for diagnostic and therapeutic procedures at the time of admission, which includes ultrasound scans as a clinical evaluation tool with minimal risk.

The SDRI was measured within the 1st 6 hours after admission to the emergency resuscitation unit. The principal investigator received standardized training from the affiliated radiologist. The initial ultrasounds were performed under the direct supervision and review of the radiologist. Subsequently, the principal investigator continued independently, but with a photographic review endorsed by the radiologist.

Additional clinical variables were simultaneously recorded using a standardized data collection tool, including demographic characteristics, vital signs, site of infection, laboratory tests, blood culture, capillary refill time, Glasgow Coma Scale, and mottling score. The occurrence of the primary outcome (in-hospital mortality) was analyzed. This research protocol was approved by the ethics and institutional committee of Fundación Clínica Shaio.

### 2.1. SDRI measurements

Ultrasound examinations were conducted using a 2.5 MHz phase-array transducer equipped with pulsed and color Doppler (Philips CX50 system; Philips Healthcare). Patients were positioned supine, and the left intercostal acoustic window was used to obtain full visualization of the spleen, and measurements were performed with color and pulsed Doppler. All ultrasound scans were performed exclusively by the principal investigator, who received prior standardized training and initial supervision from a radiologist involved in the study. A photographic record was taken, and the measurement was performed until the highest possible image quality was achieved. A detailed description of the procedure is provided in Table S1, Supplemental Digital Content, https://links.lww.com/MD/R292.

In the statistical analysis, for the description of quantitative variables, averages and standard deviation (SD) values were used after verifying their normal distribution (Shapiro–Wilk test). If normality was not identified, they were described with medians and interquartile ranges (IQRs). Qualitative variables were described by means of absolute frequencies and percentages. If normality was evidenced, the comparison of quantitative variables between groups was performed with the *Z* test for difference of means; if not, the Wilcoxon rank sum test was used. In dichotomous qualitative variables, a *Z* test for difference of proportions was used, whereas for polytomous variables, a chi-square test was used when expected values were ≥5; otherwise, a Fisher exact test was used.

In-hospital mortality was modeled using Cox proportional hazards models, checking compliance with collinearity, specificity, proportional hazards, and model fit. This model allowed us to estimate the crude hazard ratio (HR) adjusted for statistically significant confounding covariates or for variables that, according to the literature, could modify the effect. Variable selection was performed using the stepwise technique, considering an input probability of 0.1 and an output probability of 0.15. The Kaplan–Meier plot was used to compare the occurrence of the outcome of interest according to the categorized resistive index (RI).

A RI close to 1 is associated with increased vascular resistance, which could lead to compromised microcirculatory beds and hypoperfusion. Considering that there are no studies establishing a cutoff point to estimate hypoperfusion or mortality in patients with sepsis, the optimal cutoff point for the analysis was determined using several estimation models, selecting the model that generated a homogeneous distribution of patients (RI cutoff point: 0.7) (Figure S1, Supplemental Digital Content, https://links.lww.com/MD/R292). Results of the statistical tests were significant, with a *P*-value of <.05. The analysis was performed in STATA software, version 15.0 SE.

### 2.2. Definitions

- **Sepsis**: physiological, pathological, and biochemical dysregulation of the normal infection control process leading to organ dysfunction, threatening the patient’s life.^[1]^
- **Septic shock**: cellular, metabolic, and circulatory abnormalities leading to the need for vasopressor use to maintain MAP >65 mm Hg in the presence of hyperlactatemia >2 mmol/L and in the absence of hypovolemia.^[1]^
- **SDRI:** allows measurement of the movement of red blood cells (flow) to obtain distal vascular resistance,^[12]^ derived from time-velocity spectra.^[17]^ As a result, splanchnic circulation can be assessed together with alterations in arterial hemodynamics in the liver, spleen, and kidney. Formula = (peak systolic velocity − end-diastolic velocity)/peak systolic velocity.^[18]^

Missing data were analyzed as no data.

## 3. Results

**Figure 1.**
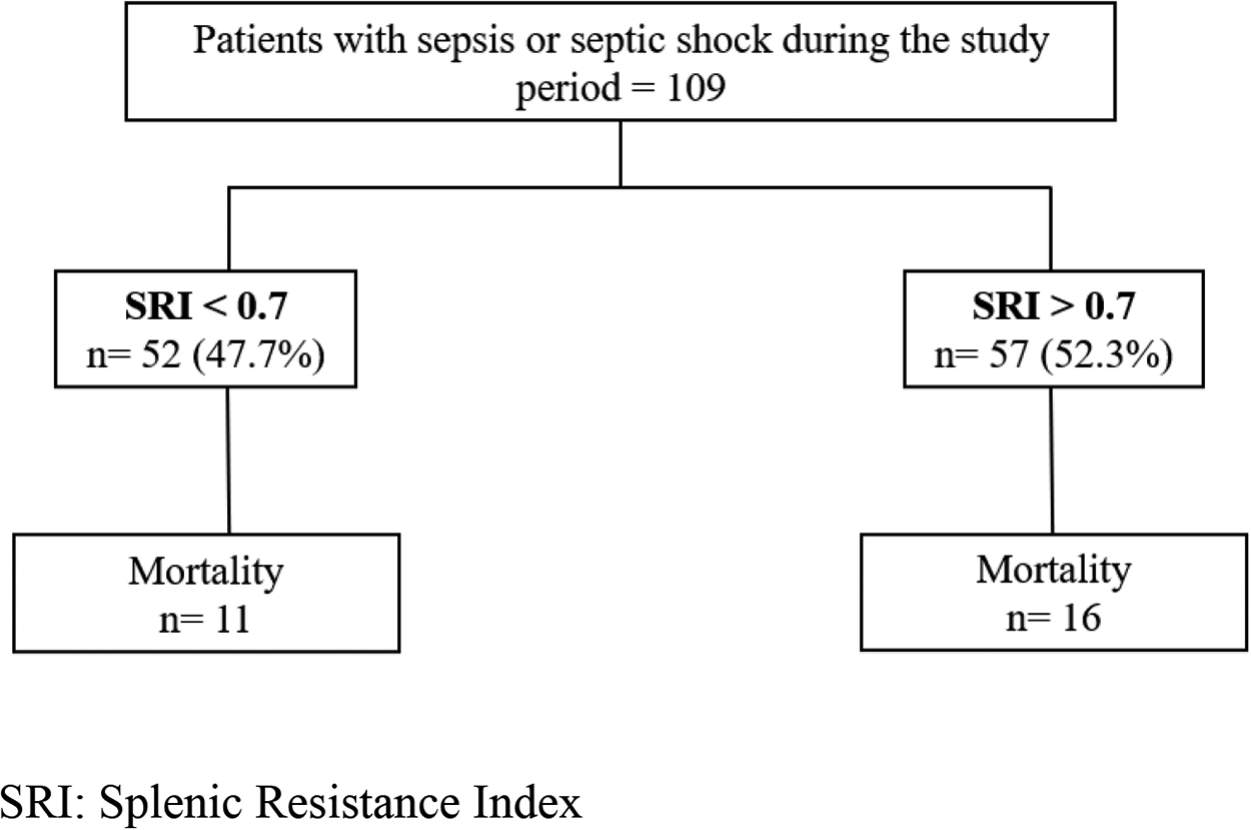
Flowchart of the participant enrollment process.

Between November 2023 and October 2024, a total of 109 patients were included in the study. Of the patients, 61% were men (n = 67) and had an average age of 75 years (IQR: 64–81 years). The most common comorbidities were arterial hypertension 52.2% (n = 57), hypothyroidism 24.7% (n = 27), diabetes mellitus 22% (n = 24), and chronic obstructive pulmonary disease 21.1% (n = 23) (Table 1). Figure 1 illustrates the flowchart of the study subjects.

**Table 1:** Sociodemographic and clinical characterization of the patients included in the study.

Variables	Global n = 109	Mortality		*P*-value
		Yes n = 27 24.77%	No n = 82 75.22%	
Age, median (IQR)	75 (64–81)	79 (61–84)	70 (64–79)	.109*
Sex, male, n (%)	67 (61.47)	16 (59.26)	51 (62.20)	.786†
History n (%)				
Diabetes mellitus	24 (22.02)	5 (18.52)	19 (23.17)	.613†
Arterial hypertension	57 (52.29)	11 (40.74)	46 (56.10)	.166†
Hypothyroidism	27 (24.77)	7 (25.93)	20 (24.39)	.873†
COPD	23 (21.1)	7 (25.93)	16 (19.51)	.479†
Coronary heart disease	17 (15.60)	6 (22.22)	11 (13.41)	.358‡
Paroxysmal AF	15 (13.76)	7 (25.93)	8 (9.76)	.051‡
Heart failure	16 (14.68)	6 (22.22)	10 (12.20)	.219‡
CKD	15 (13.76)	2 (7.41)	13 (15.85)	.349‡
Scales				
SOFA Q, median (IQR)	1 (0–2)	1 (0–2)	1 (0–1)	.191*
SOFA, median (IQR)	4.82 (2.21)	5 (4–6)	4 (3–6)	.113*
APACHE II, median (IQR)	15 (10–17)	15 (9–19)	15 (10–17)	.440*
Charlson, median (IQR)	3 (1–5)	4 (3–5)	3 (1–5)	.311*
Vital signs				
MAP, mean (SD)	82.45 (20.22)	77.77 (22.46)	84 (19.33)	.166§
HR, mean (SD)	98.72 (22.11)	98.48 (23.36)	98.40 (21.83)	.947§
RR, median (IQR)	20 (18–24)	20 (18–25)	20 (18–24)	.654*
Temperature, median (IQR)	36.4 (36–37)	36.2 (36–36.5)	36.5 (36–37.1)	.106*
Paraclinical values				
Leukocytes, median (IQR)	12,800 (8200–17,100)	13,700 (9400–17,900)	12,600 (8100–17,100)	.391*
CRP, median (IQR)	87.5 (35.1–212)	172.7 (65.8–219)	76.55 (31.6–205.5)	.280*
Creatinine, mean (SD)	1.86 (0.17)	1.73 (0.26)	1.90 (0.22)	.677§
Platelets, median (IQR)	224,000 (162,000–312,000)	218,000 (156,000–333,000)	227,000 (162,000–310,000)	.729*
Ultrasound variables				
RI < 0.7, n (%)	52 (47.71)	11 (40.74)	41 (50.00)	.403†
RI, median (IQR)	0.72 (0.64–0.79)	0.74 (0.61–0.79)	0.70 (0.65–0.79)	.841*
IP, median (IQR)	1.33 (1.13–1.57)	1.4 (1.1–1.71)	1.29 (1.13–1.57)	.885*
Gas				
Lactate, median (IQR)	1.98 (1.42–3.08)	2.67 (1.7–4.18)	1.96 (1.39–2.6)	.011*
pH, mean (SD)	7.40 (0.11)	7.38 (0.13)	7.41 (0.11)	.305§
HCO_3_, mean (SD)	18.44 (5.76)	18.23 (7.70)	18.51 (5.00)	.824§
SatO_2_, median (IQR)	91 (88–93)	92 (86–94)	91 (88–93)	.370*
PAFI, median (IQR)	280 (212–349)	288 (182–356)	277 (223–349)	.460*
Perfusion variables				
Capillary filling, mean (SD)	2.45 (0.97)	2.66 (1.30)	2.39 (0.84)	.203§
Motting n (%)				
0	91 (83.49)	20 (74.07)	71 (86.59)	.123‡
1	8 (7.34)	4 (14.81)	4 (4.88)	
2	6 (5.50)	1 (3.70)	5 (6.10)	
3	3 (2.75)	1 (3.70)	2 (2.44)	
5	1 (0.92)	1 (3.70)	0 (0)	
Glasgow, median (IQR)	15 (14–15)	15 (14–15)	15 (14–15)	.302*
Septic shock n (%)	37 (33.94)	12 (44.44)	25 (30.49)	.184†
Vasopressor n (%)	61 (55.96)	18 (66.67)	43 (52.44)	.196†
Acute kidney injury n (%)	46 (42.20)	15 (55.56)	31 (37.80)	.105†
Length of hospital stay, median (IQR)	12 (6–20)	8 (2–19)	13 (7–22)	.016*
Focus of infection n (%)				
Urinary	13 (11.93)	2 (7.41)	11 (13.41)	.776‡
Respiratory	37 (33.94)	11 (40.74)	26 (31.71)	
Abdominal∥	33 (30.28)	7 (25.93)	26 (31.71)	
Other¶	26 (23.85)	7 (25.93)	19 (23.17)	
Microbial growth, n (%)				
Gram-positive bacteria	8 (9.52)	3 (14.29)	5 (7.94)	.657‡
Gram-negative bacteria	22 (26.19)	6 (28.57)	16 (25.40)	
Mixed	1 (1.19)	0 (0)	1 (1.59)	
Negative cultures	53 (63.10)	12 (57.14)	41 (65.08)	

APACHE II = Acute Physiology and Chronic Health disease Classification System II, CKD = chronic kidney disease, CNS = central nervous system, COPD = chronic obstructive pulmonary disease, CRP = C-reactive protein, HCO_3_ = arterial bicarbonate, HR = heart rate, IP = pulsatility index, IQR = interquartile range, MAP = mean arterial blood pressure, PAFI = ratio of mean arterial oxygen pressure to fraction of inspired oxygen, RR = respiratory rate, SatO_2_ = oxygen saturation, SD = standard deviation, SOFA = Sepsis-Related Organ Failure Assessment.

* Differences estimated using the Wilcoxon rank sum test.

† Differences estimated using the chi-square test.

‡ Differences estimated using Fisher exact test.

§ Differences estimated using the *Z* test for difference in means.

∥ Abdominal focus: abdominal + gastrointestinal.

¶ Other foci: skin and soft tissues, catheter, surgical site infection, undetermined, CNS, endocarditis, myocarditis, mediastinitis, osteomyelitis, gynecological, mixed.

Scores on the SOFA, Acute Physiology and Chronic Health Evaluation II (APACHE II), and Charlson prognostic scales had an average of 4.82 (IQR: 2.21), 15 (IQR: 10–17), and 3 (IQR: 1–5), respectively. The median SOFA Q scale score was 1 in both groups (Table 1).

Non-surviving patients were characterized by lower MAP averages of 77.7 mm Hg (SD: 22.46), land temperature of 36.2°C (IQR: 36–36.5), elevated leukocyte count with a mean of 13,700 (IQR: 9400–17,900), and elevated C-reactive protein with a mean of 172 (IQR: 65.8–219). As for ultrasound assessment, we observed a higher SDRI score of 0.74 (IQR: 0.61–0.79) versus 0.70 (IQR: 0.65–0.79) and higher pulsatility of 1.4 (IQR: 1.1–1.71) versus 1.29 (IQR: 1.13–1.57). Arterial gases showed greater compromise, with values of 2.67 for lactate (IQR: 1.7–4.18) versus 1.96 (IQR: 1.39–2.6), 7.38 for pH (SD: 0.13) versus 7.41 (SD: 0.11), and 18.23 for HCO_3_ (SD: 7.70) versus 18.51 (SD: 5.0). We also observed higher incidence of septic shock with 44.44% (n = 12) versus 30.49% (n = 25), use of vasopressors with 66.67% (n = 18) versus 52.44% (n = 38), and acute kidney injury with 55.56% (n = 15) versus 37.80% (n = 31).

Capillary filling and mottling scores yielded 3 and 5 points, respectively, and did not differ between groups (2 patients in each group).

The most frequent sites of infection in non-survivors were the respiratory tract (40.74%; n = 11), the abdomen (25.93%; n = 7), and other sites (25.93%; n = 7) (skin and soft tissue, catheter, surgical site infection, undetermined site, central nervous system, endocarditis, myocarditis, mediastinitis, osteomyelitis, gynecological or mixed). In non-survivors, gram-negative bacteria were also identified more frequently in 28.57% versus 25.40% cases, followed by gram-positive bacteria in 14.29 versus 7.94% cases, with a lower percentage of negative cultures in 57.14% versus 65.08% of cases compared to survivors.

Finally, in-hospital mortality was 24.77% (n = 27). Regarding patients with RI of >0.7, 40.74% (n = 11) did not survive, and 50% (n = 41) survived, with a *P* = .403 (Table 1).

**Figure 2.**
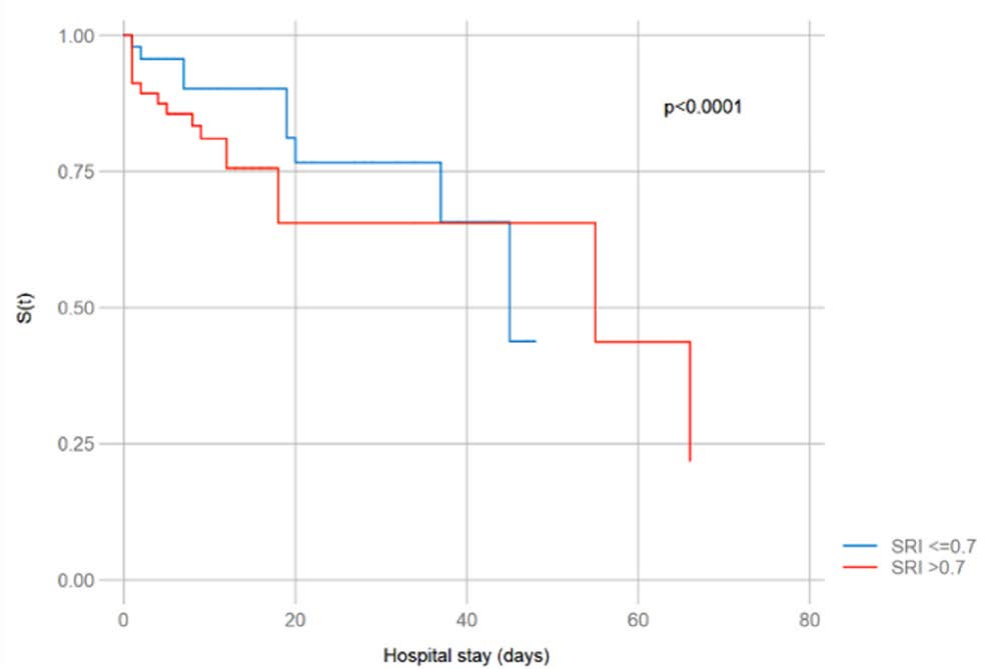
Survival analysis by Kaplan–Meier.

The Cox regression model suggests the following trend: if a patient had SDRI of >0.7, such a patient may have a 2.2 times higher risk of dying compared to patients with SDRI of <0.7, although it was not statistically significant (HR: 2.20; 95% confidence interval [CI]: 0.74–6.52; *P* = .152). In the multivariate Cox regression analysis (Table 2), several variables remained independently associated with in-hospital mortality. A lower Glasgow Coma Scale score was significantly associated with increased mortality risk (HR: 1.66; 95% CI: 1.26–2.19; *P* < .001). Similarly, longer hospital stay showed a protective association (HR: 0.60; 95% CI: 0.49–0.73; *P* < .001), indicating that non-survivors tended to have shorter lengths of stay. Oxygen saturation was also significantly associated with mortality (HR: 1.12; 95% CI: 1.01–1.24; *P* = .030), potentially reflecting persistent hypoxemia in patients who ultimately died. Additionally, the APACHE II score was positively associated with in-hospital mortality (HR: 1.15; 95% CI: 1.01–1.30; *P* = .023). Although the SDRI (SDRI > 0.7) showed a HR of 2.20, this association did not reach statistical significance (95% CI: 0.74–6.52; *P* = .152), indicating only a trend toward increased mortality. The SOFA Q score approached significance (HR: 1.72; 95% CI: 0.99–2.99; *P* = .053), suggesting a potential association that may become significant in studies with larger sample sizes. In the survival analysis, an SDRI of >0.7 was associated with reduced survival from day 0 to day 38 of hospitalization, after which survival rates between both groups converged (*P* < .0001; Fig. 2).

**Table 2:** Factors associated with in-hospital mortality in septic patients.

Variables	Raw model			Multivariate model		
	HR	95% CI	*P*-value	HR*	95% CI	*P*-value
RI > 0.7	1.53	0.65–3.57	.322	2.20	0.74–6.52	.152
O_2_ saturation	1.03	0.97–1.10	.286	1.12	1.01–1.24	**.030**
Glasgow	0.93	0.82–1.05	.264	1.66	1.26–2.19	**<.001**
Length of hospital stay (d)	0.77	0.70–0.85	<.001	0.60	0.49–0.73	**<.001**
SOFAq	1.12	0.74–1.69	.577	1.72	0.99–2.99	.053
APACHE II	1.02	0.96–1.09	.434	1.15	1.01–1.30	**.023**

The bold values indicate statistically significant results in the multivariate model, with *P*-values <.05.

APACHE II = Acute Physiology and Chronic Health Evaluation II, CI = confidence interval, GCS = Glasgow Coma Scale, HR = hazard ratio, RI = resistive index, SatO_2_ = oxygen saturation, SOFA = Sepsis-Related Organ Failure Assessment.

* Adjusted by variables: SatO_2_, GCS, length of hospital stay, SOFAq, and APACHE.

## 4. Discussion

Our study evaluated the association between SDRI and in-hospital mortality in patients with sepsis. We found that patients with SDRI of >0.7 presented lower survival in the 1st 38 days of hospital stay (*P* < .0001). Although Cox regression analysis suggested a trend toward a higher risk of in-hospital mortality in this group, this association did not reach statistical significance. These findings suggest that SDRI could be a potential marker of early hypoperfusion in sepsis, although further studies with a larger sample size are required to confirm its prognostic validity.

SDRI has been proposed as a noninvasive tool to identify occult hemorrhagic shock and predict fluid responsiveness in patients on invasive mechanical ventilation. Brusasco et al evaluated 53 patients undergoing surgery, where a change in SDRI >9% predicted fluid response with a specificity and positive predictive value of 100% (sensitivity 63%, negative predictive value 68%).^[19]^ Corradi et al analyzed 49 polytrauma patients and found that an SDRI of >0.65 (sensitivity: 0.73; specificity: 0.74; positive predictive value: 0.70; negative predictive value: 0.77; AUC: 0.90; *P* < .001) enabled the early detection of preshock and persistent hypoperfusion.^[11]^ Although the evidence on sepsis is limited, increased splanchnic vascular resistance may reflect endothelial dysfunction and microcirculatory disturbance in shock. Splanchnic circulation receives 40% of cardiac output,^[13]^ allowing up to 2/3 of its blood volume to be rapidly transferred to systemic circulation during shock.^[6,14,15]^ This process is accompanied by intense vasoconstriction (α-adrenergic receptors)^[15,16]^ as well as activation of the adrenergic system, renin-angiotensin, and vasopressin.^[5]^ The spleen, which receives 25% of cardiac output, releases approximately 100 mL of blood with high hematocrit concentrations, favoring oxygen transport.^[14]^ The spleen is the 1st to be sacrificed and the last to recover in shock states. Also, the splanchnic vasculature serves as an important blood reservoir.^[13–15]^ There is a biological plausibility supporting its study.

Despite the observed trend toward an increased risk of mortality in patients with SDRI of >0.7, the lack of statistical significance could be due to insufficient sample size or the influence of other confounding factors. Other variables, such as oxygen saturation, SOFA score, and APACHE II, showed a greater association with mortality since they are previously validated scores related to mortality and multiple organ dysfunction.^[20,21]^ However, although sepsis severity scores have proven to be useful to stratify risk and guide clinical decision making, their application is still limited due to the heterogeneity and rapid progression of sepsis.^[1]^ Therefore, there is a need to include new variables that better reflect the pathophysiology of the disease and allow an earlier and more accurate prediction of clinical deterioration.^[22,23]^ In this regard, SDRI may not be an independent predictor, but rather a reflection of overall microcirculatory dysfunction in sepsis that could support early detection of hypoperfusion.

Doppler ultrasound is an accessible and reproducible tool in the evaluation of critically ill patients.^[8]^ Its use to evaluate splanchnic perfusion could be a noninvasive alternative for the early detection of hypoperfusion.^[11,19]^ Since splanchnic hypoperfusion occurs early in sepsis, SDRI assessment by Doppler ultrasound could be a useful and noninvasive tool for risk stratification in critically ill patients. However, our findings underscore the need to standardize SDRI reference values in sepsis and to define its role within a multimodal assessment of circulatory status. Therefore, validation in prospective studies with long-term follow-up is warranted.

Our study has a few limitations. First, the sample size was relatively small, which may have limited the statistical power to detect significant associations. This is a study based on biological plausibility, and no similar studies have been conducted previously, which objectively limits the availability of reference data for accurate sample size calculation. Therefore, the sample size was estimated using the overall reported sepsis mortality prevalence (30%), yielding a required minimum of 66 patients. Our cohort included 109 patients, surpassing this threshold. Second, SDRI measurements were performed on a single occasion within the 1st 6 hours of admission, thus the dynamic evolution of the microcirculation in response to treatment was not assessed. Third, Doppler measurements were performed by a non-radiologist, but this staff member had received prior training from an expert radiologist.

Future studies should include a larger number of patients, early serial measurements of SDRI, and an analysis of its relationship with other perfusion markers such as temperature gradient, cardiac output, snuffbox resistance index, near-infrared spectroscopy, and peripheral perfusion index, which were not taken into account in our study.

## 5. Conclusions

Our findings suggest that the SDRI may be associated with reduced survival in patients with sepsis, exhibiting a trend toward increased mortality risk in those with an SDRI of >0.7. However, this association did not reach statistical significance, highlighting the need for further studies with larger sample sizes to confirm its prognostic value and generalizability.

## Acknowledgments

We would like to thank the resuscitation emergency physicians from Fundación Clínica Shaio – Fabio Garcia, Carlos Bojaca, and Juan Cardona – for their willingness and help in the realization of this project.

## Author contributions

**Conceptualization:** Tatiana Buitrago-González, Claudia Poveda-Henao, Gina Montaño-Padilla, Jenny Garzón-Ruiz, Diego Jaramillo-Hernández.

**Funding acquisition:** Tatiana Buitrago-González.

**Methodology:** Tatiana Buitrago-González, Jenny Garzón-Ruiz.

**Resources:** Diego Jaramillo-Hernández.

**Software:** Diego Jaramillo-Hernández.

**Data curation:** Tatiana Buitrago-González, Claudia Poveda-Henao, Gina Montaño-Padilla, Jenny Garzón-Ruiz.

**Formal analysis:** Tatiana Buitrago-González, Claudia Poveda-Henao, Gina Montaño-Padilla, Henry Robayo-Amortegui.

**Investigation:** Tatiana Buitrago-González, Jenny Garzón-Ruiz, Henry Robayo-Amortegui.

**Supervision:** Tatiana Buitrago-González, Claudia Poveda-Henao, Gina Montaño-Padilla, Henry Robayo-Amortegui.

**Validation:** Tatiana Buitrago-González, Claudia Poveda-Henao, Gina Montaño-Padilla, Diego Jaramillo-Hernández, Henry Robayo-Amortegui.

**Visualization:** Tatiana Buitrago-González, Claudia Poveda-Henao, Gina Montaño-Padilla, Diego Jaramillo-Hernández, Henry Robayo-Amortegui.

**Writing – original draft:** Tatiana Buitrago-González, Claudia Poveda-Henao, Gina Montaño-Padilla, Jenny Garzón-Ruiz, Henry Robayo-Amortegui.

**Writing – review & editing:** Tatiana Buitrago-González, Claudia Poveda-Henao, Gina Montaño-Padilla, Henry Robayo-Amortegui.

## Supplementary Material


