# Supplementary material for: Evaluation of the splenic Doppler resistive index as a hemodynamic variable and its association with mortality in sepsis: A prospective cohort study
Source: Medicine (Baltimore). 2026 Jan 30;105(5):e47433. doi: 10.1097/MD.0000000000047433 (PMC12863860; doi:10.1097/MD.0000000000047433)
Supplement: Supplementary file 1 [file medi-105-e47433-s001.docx]

**Supplementary Material**

**Supplement Figure 1. Splenic resistance index and perfusion**


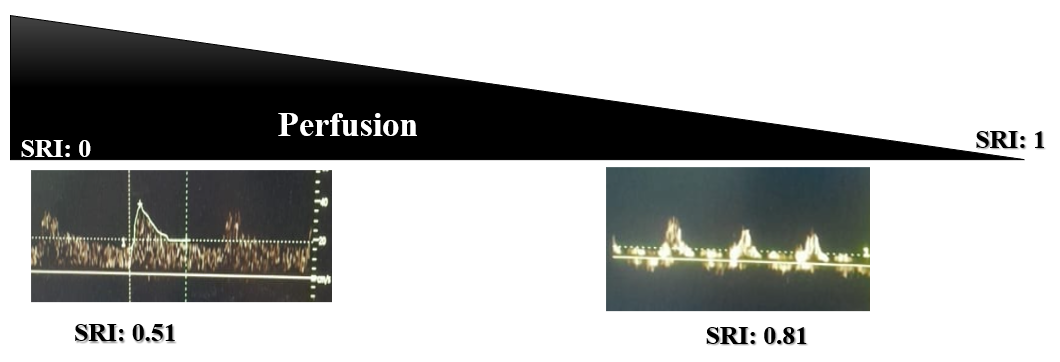


SRI: Splenic Resistance Index

**Supplement Table 1. Technical considerations for splenic Doppler**

| **Initial considerations:**   1. Review the patient’s medical record to assess inclusion and exclusion criteria. Review if the patient has previous imaging available (ultrasound, CT, or abdominal MRI), taking note of reported abnormalities in the spleen and splanchnic circulation. 2. Verify the patient’s identity (full name and identification document). 3. Introduce yourself to the patient 4. Request verbal informed consent for acceptance of participation in the study. 5. Explain how the procedure will be performed. |
| --- |
| **Technical considerations:**   1. Position the patient in right lateral decubitus, if tolerated, given their hemodynamic status, or otherwise in the supine position. 2. Turn on the ultrasound machine, select the abdominal scan software, and use the convex transducer. In the left hypochondrium, below the costal ridge or between the intercostal spaces (intercostal window), locate the splenorenal space. Ensure complete visualization of the spleen. Directed deep breaths may be necessary. 3. Activate the color Doppler tool to identify the main branches of the splenic artery, 1 cm from the hilum, in a straight path of the vessel, avoiding peripheral and tortuous vessels. 4. Gain and frequency should be adjusted to obtain an optimal color image, avoiding artifacts due to overflow or *aliasing*. Select the lowest frequency range that does not produce artifacts in the evaluated vascular structure, and adjust the wall filter to the lowest possible setting. 5. Subsequently, using pulsed Doppler, the peak systolic velocity (PSV)—the maximum flow velocity recorded during each cardiac cycle—and the end-diastolic velocity (EDV)—the velocity immediately preceding the systolic upstroke—will be measured, and the resistive index (RI) will be calculated automatically. The RI is calculated using the following formula: RI = (VPS − [VTD / VPS]). The Doppler gate should be adapted according to the lumen of the vessel to be explored. The angle of incidence of the ultrasound beam should always be less than or equal to 60°. 6. According to the Doppler theory, the flow velocity will be calculated with the following formula: V = (Fd − C) / (2Fo − W), where Fd is the frequency of flow displacement in Doppler analysis, C is the speed of sound displacement in soft tissues (approximately 1540m/s), Fo is the frequency of the emitted sound wave and W is the angle between the ultrasound beam and the longitudinal axis of the vessel. 7. Photographic recording of 2D and pulsed Doppler images will be performed throughout the process. |

**Supplement Table 2: Macrovascular and microvascular hypoperfusion variables**

|  | Advantages | Disadvantages |
| --- | --- | --- |
| CLINICAL | | |
| Capillary filling | - Suitable for ICU  - Good correlation with lactate and urine output.  - Clinical visualization of local microcirculation.  - Reproducible in septic shock  - High concordance between evaluators  - May be related to splanchnic hypoperfusion  - Correlates with pulsatility index  - Associated with high SOFA  - Prognosis in septic shock  - Associated with in-hospital mortality | - Has a standardized protocol for proper measurement.  - Does not reflect cardiac output  - Reproducibility controversies  - Varies by pressure application site, ambient and skin temperature, gender, age, ambient light, etc. |
| Mottling score | - Easy to perform  - Good correlation with lactate and urine output.  - High PPV for mortality in septic shock.  - May be related to splanchnic hypoperfusion  - Organic failure severity  - Allows to determine prognosis | - Difficult to assess in the black race.  - Should be differentiated from autoimmune disease  - Depends on the observer |
| MAP | - May reflect cellular hypoperfusion. | - Non-pathognomonic finding. |
| Temperature gradient | - Evaluates peripheral perfusion  - Measures peripheral vasoconstriction  - Associated with poor prognosis in abdominal surgery. | - Affected by room temperature. |
| GASIMETRIC | | |
| Arterial lactate >2 | - Global perfusion parameter  - Widely used in clinical practice  - Marker of hypoperfusion and tissue morbidity and mortality in shock  - >4 increases probability of microcirculatory anomalies  - Cell perfusion surrogate | - Does not directly represent microcirculation  - Does not determine regional or occult perfusion  - Lower sensitivity and specificity  - Affected with liver and kidney dysfunction and drugs |
| Venous saturation <70% | - Reflects balance between O_2_ transport and consumption in global arterial saturation.  - Optimizing this parameter within the first 6 h of resuscitation improves prognosis in septic shock | - Does not represent direct evaluation of microcirculation.  - Controversy in the prediction of microcirculatory abnormalities in sepsis.  - May be normal despite hypoperfusion |
| Delta CO_2_ <6 | - Reflects low cardiac output with hypoperfusion.  - Mitochondrial dysfunction marker  - Indicates decreased systemic blood flow.  - Useful for assessing perfusion in septic shock  - Microvascular dysfunction marker | - Depends on factors that modify Hb CO_2_ and CO_2_  production |
| ULTRASOUND | | |
| Echocardiography | - Indirect measurement of vascular tone.  - Longitudinal deformation of the left ventricle could help in the early detection of myocardial ischemia due to hypoperfusion.  - It is associated with hyperlactatemia and low venous saturation in septic shock. | No evidence of organ-specific hypoperfusion |
| Cardiac output | - Main determinant of DO_2_  - Recommended by Surviving Sepsis Campaign | No evidence of organ-specific hypoperfusion |
| Renal Arterial Doppler Resistive Index | - Fast and noninvasive tool  - Real organ perfusion reflex and systemic vascular hypoxia.  - Predicts acute kidney injury in sepsis.  - The higher its value, the greater the supply-demand imbalance must be ruled out.  - May reflect early vascular response.  - Increased sensitivity in splenic occult hypoperfusion due to hypovolemia.  - High predictive values for hemorrhagic shock in polytrauma.  - Good feasibility and confidentiality for inexperienced operators.  - Microcirculatory parameter. | - Not useful in determining the cause of hypoperfusion.  - Lack of generalization in the ICU |
| Snuffbox Resistance Index | - Determines the state of the peripheral vasculature in critical patients.  - Correlates with IP and lactate clearance | - Not useful in determining the cause of hypoperfusion.  - Lack of generalization in the ICU |
| OTHER | | |
| Sublingual partial pressure of carbon dioxide (PslCO_2_) | - Directly related to gastric carbon dioxide partial pressure.  - Good indirect indicator of splanchnic microcirculation | - Research tool.  - Expensive and impractical microscopes.  - It is unclear whether the sublingual microvascular bed reflects other territories such as intestinal mucosa |
| NIRS (near infrared spectroscopy) | - Evaluates oxygen consumption indirectly  - Tissue saturation specific to organs  - Low thenar oxygen saturation in polytrauma patients correlates with multiple organ failure  - Low saturation in septic shock |  |
| Peripheral perfusion index | - Provides information on peripheral vascular tone by pulsatility, decreased vasoconstriction and increased vasodilatation  - Early predictor of central hypovolemia in critically-ill patients  - <0.3 predicted vasopressor therapy and less than 0.2 predicted mortality  - <1.4 hypoperfusion marker  Noninvasive numerical measurement of peripheral perfusion | - High interindividual variability |
